# 1418. Single Center Treatment Patterns for Asymptomatic Bacteriuria and UTIs in Kidney Transplant Recipients: Are We Still Overtreating?

**DOI:** 10.1093/ofid/ofab466.1610

**Published:** 2021-12-04

**Authors:** Meena Azeem, Kelsie Cowman, Cindy Pynadath, Rachel Bartash

**Affiliations:** 1 Montefiore Medical Center, Bronx, New York; 2 Montefiore Medical Center and Albert Einstein College of Medicine, New York, NY; 3 Montefiore Medical Center/Albert Einstein College of Medicine, Bronx, New York

## Abstract

**Background:**

In February 2019, the American Society of Transplantation (AST) published guidelines on the management of asymptomatic bacteriuria (AB) and urinary tract infections (UTIs) in kidney transplant (KT) recipients. These recommendations include avoiding treatment of AB > 2 months post-transplant and outline the duration of treatment (DT) for uncomplicated and complicated UTIs. We reviewed management of these syndromes and guideline concordance at our institution.

**Methods:**

We conducted a single-center, retrospective cohort study of KT recipients age > 18 years who underwent transplantation between June 2016 - June 2020. Patients were obtained through query of our electronic medical record for documented UTI syndromes and included if a diagnosis was confirmed between March 2019 - December 2020 upon chart review. Definitions of AB, complicated UTI, and uncomplicated UTI were based on AST definitions. Patients with AB < 2 months post-transplant were excluded. Outcomes included treatment of AB, DT, 30-day hospital admission and re-admission, and 30-day mortality. Bivariate analysis was conducted using chi square and t test.

**Results:**

Seventy-four patients (mean age 55.4 years, 62.0% female) were included. Twenty-one patients had AB, 90% of whom received antibiotics. Distribution of diagnoses and median DT among those treated with antibiotics is outlined in Table 1.

Overall DT was similar in patients whose care included infectious disease (ID) input and those who did not (10.0 vs. 10.0 days, p=0.12), although ID involvement was more common in complicated UTIs. There was no difference in 30-day admission rates for those receiving < 7 days vs. > 7 days of antibiotics (p=0.53) (Table 2) including those with complicated UTIs (5/15 in < 7 days (33%) vs. 4/18 in > 7 days (22%) p=0.49). There were no deaths within 30 days of diagnoses.

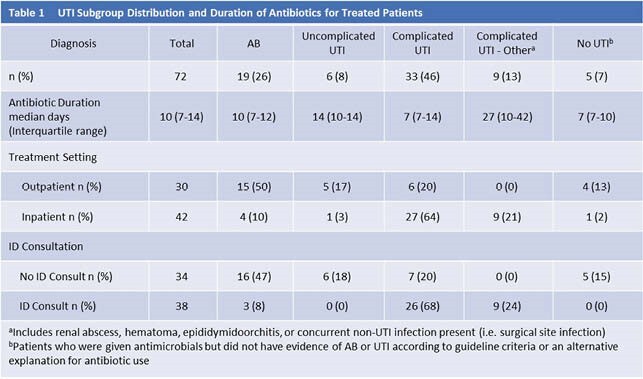

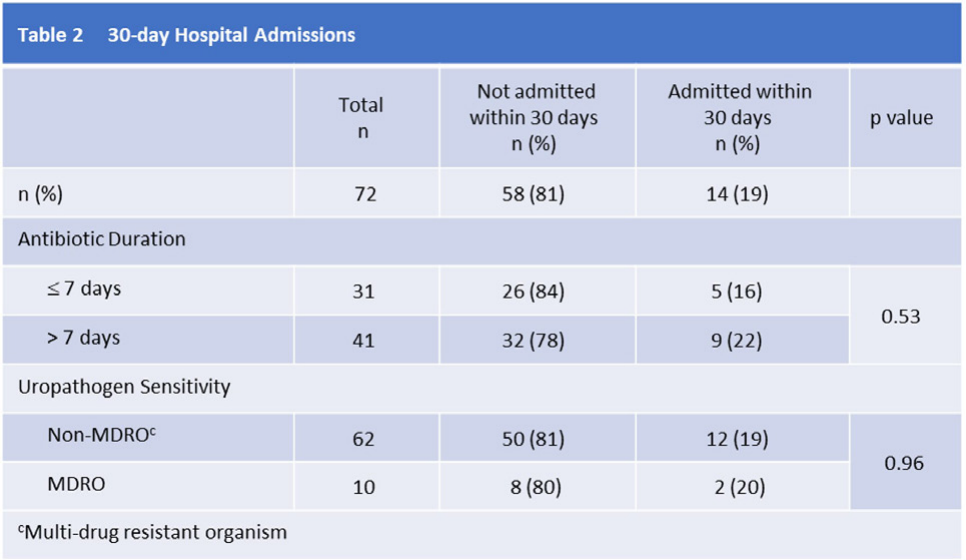

**Conclusion:**

Despite guidelines, treatment of AB is common and uncomplicated UTIs often receive prolonged courses of antibiotics. There was no increased risk of admission or mortality with shorter DT, though these results should be interpreted cautiously given the small sample size. Greater stewardship efforts are needed in this high-risk population.

**Disclosures:**

**Kelsie Cowman, MPH**, **Merck** (Research Grant or Support)

